# Perceived leader integrity as a mediator between ethical leadership and ethical climate in a teaching context

**DOI:** 10.1186/s40359-020-00420-6

**Published:** 2020-05-20

**Authors:** Ibeawuchi K. Enwereuzor, Ike E. Onyishi, Florence Chiji Albi-Oparaocha, Kenneth Amaeshi

**Affiliations:** 1 Department of Psychology, University of Nigeria, Nsukka, Nsukka, 410001 Nigeria; 2 College of Medicine, Alex Ekwueme Federal University, Ndufu-Alike, Ikwo, P. M. B. 1010, Abakaliki, Ebonyi State Nigeria; 3 University of Edinburgh Business School, University of Edinburgh, 29 Buccleuch Place, Edinburgh, EH8 9JS UK

**Keywords:** Ethical climate, Ethical leadership, Head teacher, Leader, Organisation, Perceived leader integrity, School, Teaching context

## Abstract

**Background:**

Scandalous incidents occurring in prominent organisations in the world have brought to limelight the role of leaders in shaping the ethical climate of their organisations. As a result, several studies across different organisational/occupational contexts and climes have examined and unanimously proven that ethical leadership was positively related to ethical climate. However, there is rarely any of these studies that was conducted in teaching context. Besides, the mechanisms involved between ethical leadership and ethical climate seems not to have been addressed in literature. Thus, this paper reports the findings of a study that investigated the mediating role of perceived leader integrity in the ethical leadership–ethical climate relationship among teachers.

**Methods:**

Data were collected from 336 teachers (105 male and 231 female) in three-time periods using measures of ethical leadership, perceived leader integrity, ethical climate, and demographics.

**Results:**

The results from OLS regression-based path analysis showed that: 1) ethical leadership was positively related to perceived leader integrity, 2) perceived leader integrity was positively related to ethical climate, 3) ethical leadership was positively related to ethical climate, and 4) the positive relationship between ethical leadership and ethical climate was mediated by perceived leader integrity.

**Conclusions:**

The current study extends the social learning theory by identifying perceived leader integrity as a mechanism underlying the relationship between ethical leadership and ethical climate. The findings have some implications for personnel selection especially in relation to selection of ethical leaders.

## Background

The attention of the general public, researchers, and other stakeholders have been drawn to the inherent dangers of dubious organisational practices following the scandalous incidents that occurred in organisations such as Enron, WorldCom, Adelphia, Siemens, Tyco International and the like. These incidents brought to the fore the issue of ethically questionable behaviour in the corporate environment and also suggest that unethical behaviour may be one of the probable major issues confronting the contemporary world of work. Unfortunately, it has equally been argued that some organisations are so engrossed with meeting their performance goals at the detriment of taking into consideration the ethical aspect of accomplishing such goals [3, 13]. Evidence in support of this stance comes from various forms of unethical practices that have been reported in organisations across countries (for details, see [2, 62]). For example, Hamilton and Gabriel [23] identified some fraudulent practices perpetrated by organisations in Nigeria to include funds diversion, secret commission and bribery, false invoicing, theft of inventory assets, and cheque forgery.

Nigeria is a West African country made up of 36 states and the Federal Capital Territory (FCT) located in Abuja. She is regarded as the most populous in Africa. She is a member of the Organisation of Petroleum Exporting Countries (OPEC), and regarded as one of the major crude oil producers in the world, which constitutes the major source of foreign exchange revenue for the country. However, in spite of being an oil-rich country and bestowed with many natural resources, she is still amongst the poorest in the world. Recently, she was pronounced by the Brookings Institution based on data from the World Poverty Clock as the world’s poverty capital, having the highest number of people living in extreme poverty (see [59]). Furthermore, she has consecutively been ranked among the most corrupt countries in the world (see [51–55]).

The Economic and Financial Crimes Commission (EFCC) and Independent Corrupt Practices and other Offences Commission (ICPC) are the two anti-graft agencies saddled with the responsibility of arresting and prosecuting persons involved in embezzlement of public funds in the country. Although, in the teaching/education context, offences such as cheating during examinations, impersonation, forgery of result slip, and stealing of question papers, among others, attract a fine of minimum of ₦50,000 and maximum of 5 years imprisonment as enshrined in the Examination Malpractice Act No. 33 of 1999, however, it is not devoid of unethical practices. For instance, in 2017, the Joint Admissions and Matriculation Board (JAMB) blacklisted 72 out of the 600 Computer-Based Test (CBT) centres for their involvement in examination malpractice in the 2017 Unified Tertiary Matriculation Examination (UTME; see [39]). In that same year, JAMB also recorded 2508 cases of examination malpractice which, however, dropped to 208 in 2018 (see [42]). Similarly, the West African Examinations Council (WAEC) in its 65th National Examinations Committee meeting decried the increasing rate of collusion to perpetrate examination malpractice which led to the cancellation of entire results (CER) or cancellation of specific subjects of some candidates. Some candidates were also barred from participating in the council’s examinations for a number of years [24]. All in all, these call into question the ethical climate of the teaching/education context of Nigeria which calls for scholarly attention.

Ethical climate refers to an aspect of organisational climate that represents the holistic impression of employees concerning the content and extent of the prevalent values, norms, attitudes, and behaviours of the organisational members [5] as it pertains to ethics. Organisational values that concern ethical issues, as well as those that stipulate what are regarded as ethically acceptable behaviour, constitutes the ethical climate of an organisation [61]. In other words, it involves the shared perceptions of what ethically correct behaviour is and how ethical issues should be addressed [37] in the workplace. Through formal and informal socialisation process in an organisation, employees learn how to behave. They become aware of the values that are upheld and rewarded in the organisation and the ones that are unacceptable [61].

Notwithstanding that ethical climate has been extensively studied in a variety of organisations including technology, insurance, hotels and restaurants, accounting and financial, legal, and medical organisations, among others (e.g., [17, 34, 35, 60]), only few studies have been conducted in educational settings (e.g., [4, 47, 48]). However, these studies were conducted outside the Nigerian teaching/educational setting which may differ from that of Nigeria.

As Van Aswegen and Engelbrecht [60], and Sağnak [47] point out, organisational leaders play an important role in determining the ethical climate of an organisation. When faced with ambiguous ethical climate and ethical dilemmas, subordinates often turn to their leaders for guidance and direction [11, 28]. In teaching context for example, if the head teacher as a leader is seen as someone who condones unethical practices, then the rest of the teachers (subordinates) may also decide to engage in unethical practices such as aiding the pupils/students in engaging in examination malpractice in exchange for money or other benefits from the parents. In this respect, leaders cannot be completely exonerated from shaping the ethical (or unethical) climate of an organisation.

However, ethical aspect of leadership has been mostly unexplored, even when it offers great avenues for novel discoveries [10]. Given that research on ethical leadership is just emerging [36], only limited number of studies have been conducted on the link between ethical leadership and ethical climate (e.g., [16, 35, 41, 49]). As such, Mayer et al. [36] advocate for research on the link between ethical leadership and ethical climate to be conducted. However, most of the studies that have paid heed to the call so far were conducted outside teaching context.

While these studies seem to have sufficiently proven that ethical leadership promotes ethical climate, a key question, however, that is yet to be answered in literature to our knowledge is *how* ethical leadership promotes ethical climate. To address this issue, the current study, therefore, attempts to identify a probable mechanism underlying the relationship between ethical leadership and ethical climate in teaching context. In that sense, this study attempts to open the black box of the link between ethical leadership and ethical climate by proposing perceived leader integrity as a mechanism that can facilitate the relationship between head teachers’ ethical leadership style and the perception of ethical climate by subordinate teachers.

Although a previous study by Van Aswegen and Engelbrecht [60] have attempted to examine the moderating role of perceived leader integrity in the relationship between transformational leadership and ethical climate, no studies that we are aware of have explicitly examined the mediating role of perceived leader integrity in the relationship between ethical leadership and ethical climate. This study thus examines a model in which the influence of ethical leadership on ethical climate is mediated by perceived leader integrity among teachers. The rationale for teachers in this study was because they are at the forefront of educating students. Therefore, they have great influence on the lives of many individuals who pass through them throughout their teaching career. They also serve as role models and mentors to their students who look up to them for guidance and direction in life. Therefore, how ethically compliant teachers are may reflect in the ethical conduct of their current and former students. Hence, understanding the role of leadership in ethics among teachers appears worthwhile. Besides, there is rarely any study of this nature that has been conducted in teaching context. The results of this study have the potential to provide valuable information not only to school management and teachers but also to other stakeholders in the education sector.

## Theoretical foundations and hypotheses development

Social learning theory serves as a theoretical foundation for contending that ethical leadership will engender ethical climate in teaching context. Social learning theory proposes that individuals are influenced by observing role models in their environment [7]. According to Bandura [7], almost anything that can be learned through direct experience can also be learned by vicarious experience, through observing other peoples’ behaviour and its attendant consequences. Such consequences make it easy to learn in an anticipatory way.

In work setting, employees can learn what type of behaviours are accepted, commended, and penalised through role modelling. Thus, they become informed about the advantages of the modelled behaviour and the disadvantages of improper behaviour. For a person to be regarded as a role model, the person must be seen by others as credible and attractive. Being seen as credible and attractive are hinged on the power and status of the individual in question [7]. When those that are looked at by others as likely role models occupy high status or powerful position, others will attempt to emulate their behaviour because it expresses expectations and approved norms [7]. A leader such as a head teacher is a significant and possible source of such role model due to their assigned role, high standing status in a school, and their positional power to influence the behaviour of other teachers to accomplish schoolwork-related outcomes.

A social learning viewpoint on ethical leadership will suggest that ethical leaders (head teachers) influence the ethical behaviour of their subordinates (i.e., other teachers who occupy lower cadre in the school) through modelling. Thus, if head teachers as leaders are to be viewed as ethical leaders who can affect their subordinates’ ethical conduct, they must first of all demonstrate exemplary credibility in their own conduct as role models because other teachers may be suspicious about ethical assertions made by such leaders. A head teacher becomes attractive and credible as an ethical role model by engaging in behaviours that are appraised by subordinates as ethical.

Therefore, ethical leaders become social learning models by rewarding proper conduct and meting out punishment for misconduct [56]. By setting the ethical tone of a school and providing a road map to guide the ethical conduct of the subordinates, such leaders are likely to be perceived by the subordinates as leaders who maintain high level of integrity in discharging their leadership responsibilities. In turn, such perception of leader integrity may cascade to the ethical climate of the school. In other words, teachers are likely to see ethical head teachers as those with integrity which, over time may contribute to the formation of ethical climate of the school. Thus, we extend the social learning perspective by incorporating perceived leader integrity as a potential mechanism that helps transmits the influence of ethical leaders in a teaching context. That is, ethical leadership will lead to perception of leader integrity which subsequently will lead to perception of being surrounded by high level of ethical climate in the school. Previous studies based on social learning theory have provided support for this theory especially in the areas of ethical leadership and ethical climate (e.g., [35, 49]) as well as perceived leader integrity [44] and their links to important organisational outcomes.

### Ethical leadership and ethical climate

Grojean, Resick, Dickson, and Smith [22] assert that besides enhancing organisational efficiency, leaders equally have the responsibilities of guiding the behaviours of their subordinates and institutionalising the ethical values and conduct of members of the organisation. One style of leadership that seem to align themselves with these responsibilities is ethical leadership given that they strive to convey high ethical values to their subordinates. Accordingly, ethical leadership refers to *the demonstration of normatively appropriate conduct through personal actions and interpersonal relationships*, *and the promotion of such conduct to followers through two-way communication*, *reinforcement*, *and decision-making* [italics in original] ([11], p. 120). Behaving in a normatively appropriate way means to behave in accordance with general expectations on how leaders ought to behave in a corporate environment. This suggests that leaders should be honest, fair, trustworthy, and caring and answerable for their conduct, as well as to reward and punish subordinates accordingly in order to hold them accountable for their actions.

By acting as role models of normatively appropriate conduct and using reward and punishment to encourage ethical conduct [11, 56], ethical head teachers signal to the subordinates (i.e., lower-cadre teachers) that nothing short of doing the right thing is expected from them and valued by the organisation. In no time, the lower-cadre teachers are more likely to perceive an ethical climate in their school. Consistent with this view, Grojean et al. [22] acknowledge that even though other factors might contribute to the determination of ethical climate, leaders exert the greatest influence on the ethical climate of an organisation.

Lending support to the above argument, Neubert et al. [41] collected Internet-based data from full-time employees and found a positive relationship between ethical leadership and ethical climate. Mayer et al. [35] examined the mediating role of ethical climate in the ethical leadership–employee misconduct relation among employees from a variety of organisations in the United States. Mayer et al.’s results show that ethical leadership was positively related to ethical climate, and that ethical climate mediated the relationship between ethical leadership and employee misconduct. Similarly, in South Korea, Shin [49] found a positive relationship between chief executive officers’ (CEOs’) self-rated ethical leadership and employees’ aggregated perceptions of the ethical climate of the organisation. In similar vein, Lu and Lin [32] also found positive relationship between ethical leadership and ethical climate based on data collected from employees of Taiwan International Ports Corporation (TIPC) in Taiwan. Demirtas and Akdogan [16] examined the indirect relationship between ethical leadership and organisational outcomes (i.e., affective commitment and turnover intention) through ethical climate. The participants involved middle-level managers, engineers, chiefs of the maintenance shops, and blue-collar full-time employees of aviation industries. The results show that ethical leadership was positively related to ethical climate. Ethical climate partially mediated the relationship between ethical leadership and affective commitment. Also, ethical climate partially mediated the relationship between ethical leadership and turnover intention. More recently, Al Halbusi, Williams, Mansoor, Hassan, and Hamid [1] found that ethical leadership was positively related to employees’ ethical behaviour in Baghdad (Republic of Iraq).

In sum, the above studies have unanimously provided strong evidence showing that ethical leadership has direct positive relationship with ethical climate across diverse climes and organisational/occupational contexts but not teaching. Moreover, most of these studies were conducted in the United States and Asia with their own cultural peculiarities. As such, research is yet to ascertain whether similar findings would be obtained in Nigerian teaching context. Besides, one key question that remains to be addressed in literature bothers on *how* ethical leadership influences ethical climate. That is, what mechanism is involved in the ethical leadership-ethical climate relationship? Asking such a question is very important because according to Baron and Kenny [8], when there is such consistency in the relationship between two variables, then it is likely that there is a mediator between them that tend to facilitate the relationship. Accordingly, in response to that question, we propose perceived leader integrity as a mechanism (i.e., mediator) underlying the link between ethical leadership and ethical climate. As such, the current study complements the literature in this area by opening the black box behind the ethical leadership-ethical climate relationship.

### Perceived leader integrity as a mediator

Perceptions of leader integrity has been identified as a fundamental characteristic of effective leadership [40, 43]. Such perceptions are important to followers because they embody important information which helps in minimising the incertitude surrounding the decision to follow [30]. When a leader is seen by followers as having integrity, they become confident that the leader will lead in honest, felicitous, and concordant manner in line with professed vision [40]. Given the importance of leader integrity in subordinates’ impression about the leader, we opted to assess leader’s integrity as perceived by subordinates which we reckon, could help subordinates in forming holistic impression about their organisation.

Perceived leader integrity, according to Craig and Gustafson [15], refers to employees’ perception of the moral behaviour demonstrated by their leader. This suggests that perception of leader integrity elicit a judgment that the leader is seen as a moral person. Therefore, in order to be effective, leaders should be perceived by their followers as exhibiting a level of integrity in accordance with followers’ expectations [15]. According to Badaracco and Ellsworth [6], leaders with integrity will strive to be consistent in whatever they believe in, how they lead others, and the type of organisations they want to identify with. They keep to their words even when it appears to be inconvenient to do so [11]. Such leaders usually cultivate open and honest communication especially in discussions that are related to decision-making [60]. As Malan and Smit [33] stated, leaders are committed and loyal to the organisation when they lead with integrity and demonstrate what consistency in behaviour means in line with what they say. Consistency in terms of decisions and behaviour will make a leader to be seen as dependable, trustworthy and having integrity [18, 46].

In a teaching context, teachers are likely to perceive their head teacher who adopts ethical leadership style as also having integrity. This is because by being cautious of ethical standards and paying close attention to the consequences of personal and organisational decisions on ethical issues, ethical leaders signal to subordinates the importance and value attached to integrity. Honesty, fairness, trustworthy, and integrity [11, 46, 57] appear to be the watch word of an ethical leader. Thus, adopting ethical leadership is likely to make subordinates perceive such a leader as someone with high integrity. In turn, subordinates may come to view themselves as being surrounded by ethical climate in their school. Consistent with this view, Litwin and Stringer [31] observed that the understanding of realities in organisational setting is hinged on the perception of the organisational members. In that sense, perceiving a head teacher as having integrity may be an important mechanism through which ethical leaders promote the ethical climate of their school.

Existing studies can be used to draw inference for our arguments regarding the role of perceived leader integrity. For instance, one study indicated that integrity moderated the relationship between certain aspects of transformational leadership and some aspects of ethical climate among employees of medium and large organisations in South Africa (e.g., [60]). In another study, it was reported that organisational justice mediated the positive relationship between ethical leadership and employees’ ethical behaviour [1]. In addition, Neubert et al. [41] found that interactional justice moderated (boosted) the relationship between ethical leadership and ethical climate among full-time employees based on Internet data. In addition, Schminke et al. [48] found that leader Utilizer score (U-score or moral development utilization), which refers to the consistency between the leader’s moral development and actions, moderated the relationship between leader moral development and organisational ethical climate such that the relationship between leader moral development and ethical climate was stronger for high U-score leaders than for low U-score leaders.

**Fig. 1:**
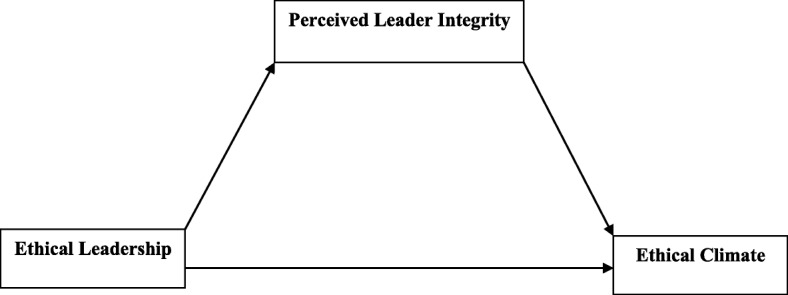
Hypothesized model of perceived leader integrity as a mediator between ethical leadership and ethical climate

Given the promising empirical support for perceived leader integrity, one would expect that ethical leadership will be related to perceived leader integrity which in turn is likely to mediate the ethical leadership–ethical climate relationship among teachers. See Fig. 1 for the hypothesized model of the current study.

## Hypotheses

- *Hypothesis 1*: Ethical leadership will be positively related to perceived leader integrity.
- *Hypothesis 2*: Perceived leader integrity will be positively related to ethical climate.
- *Hypothesis 3*: Ethical leadership will be positively related to ethical climate.
- *Hypothesis 4*: The positive relationship between ethical leadership and ethical climate will be mediated by perceived leader integrity.

## Method

### Participants

Three hundred and thirty-six (336) teachers (with an average of 18 teachers per school) from 19 primary schools participated in this study. The small number of teachers makes it possible for each head teacher to be able to interact directly with the rest of the teachers in each school without any hierarchical leadership structure in-between them. The participants comprised 105 (31.3%) male and 231 (68.8%) female teachers. In terms of age, 41 (12.2%), were less than 25 years, 205 (61.0%) were between the ages of 25 and 40 years, 73 (21.7%), were between the ages of 41 and 56 years, while 17 (5.1%) were above the age of 56 years. The married ones among them were 217 (64.6%) while the single ones were 119 (35.4%). With regards to job position, 94 (28%) were junior staff while 243 (72%) were senior staff. Of these participants, four (4) (1.2%) of them had teacher training certificate, 10 (3.0%) had senior secondary school certificate, 109 (32.4%) had an ordinary national diploma (OND) or a national certificate of education (NCE), 163 (48.5%) had a highest national diploma (HND) or a bachelor degree, and 50 (14.9%) had a postgraduate degree.

### Measures

#### Demographics

We asked participants to provide information about their gender, marital status, age, tenure with current organisation, and highest educational qualification with which we created their demographic profile.

#### Ethical leadership

We assessed ethical leadership with the 10-item unidimensional Ethical Leadership Scale (ELS; [11]). The ELS appears to be the mostly used scale in research for assessing ethical leadership with well-established psychometric properties (e.g., [1, 16, 41, 49]). Respondents rated each item on a 5-point scale ranging from 1 (*strongly disagree*) to 5 (*strongly agree*) to indicate the degree to which each statement describes their head teacher. Sample item includes: “Makes fair and balanced decisions”. A respondent can obtain a possible score that ranged from 10 to 50. Higher scores indicate that their head teacher employs greater ethical leadership style. A high Cronbach’s alpha (α) of .89 obtained both in the current sample and in a recent study in Nigeria by Enwereuzor, Adeyemi, and Onyishi [19] is comparable to the .92 reported by Brown et al. Together, these indicate that the scale is highly reliable by surpassing the threshold of .70 recommended for research purpose [27]. This explains the choice of the ELS in the current study.

#### Perceived leader integrity

Perceived leader integrity was assessed with the 31-item Perceived Leader Integrity Scale (PLIS; [15]). The PLIS is an established means of assessing perception of leader integrity and widely used in research (e.g., [14, 38]), which justifies its use in the current study. Respondents rated each item on a 4-point scale ranging from 1 (*not at all*) to 4 (*exactly*) to indicate how well each item describes their immediate head teacher. Sample item include: “Would use my mistakes to attack me personally”. The items on the scale are negatively worded. Hence, we reverse scored them such that higher scores represent perceptions of higher leader integrity. A possible score on this scale ranged from 31 to 124. Craig and Gustafson reported Cronbach’s α of .97. Similarly, high level of Cronbach’s α of .89 was also reported in a United States sample [14]. We obtained a comparable Cronbach’s α of .90 in the current sample, which exceeds the minimum benchmark of .70 required for research purpose [27]. The Cronbach’s α value obtained in the current sample indicates that the scale is highly reliable.

#### Ethical climate

We assessed ethical climate using the 19-item Ethical Climate Index Short Form developed by Arnaud [5]. The scale was designed to assess ethical climate. Respondents rated each item on a 5-point scale ranging from 1 (*strongly disagree*) to 5 (*strongly agree*) to indicate the degree of their agreement or disagreement with statement such as “People in my department recognize a moral dilemma right away”. A possible score on this scale ranged from 19 to 95. Higher scores indicate greater perception of organisational ethical climate. Arnaud reported Cronbach’s α ranging from .80 to .90 for each of the dimensions of the short form. The Cronbach’s α for the overall ethical climate in the current study was .79, which compares well with a recent study that reported .93 [20] and indicates that the scale is reliable, exceeding the minimum threshold of .70 stipulated for research purpose [27]. The popularity of the scale and established psychometric properties explain why the scale was used in the current study.

#### Procedure

We invited teachers to participate in the current study through a cover letter that was approved by the head teacher of each of the schools that participated. The cover letter provided explanations on purposes of the study, assurance of confidentiality of responses, encouraged the participants to be honest in their responses, and informed them that the data would be used for research purposes only. Specifically, they were informed to report their ages in ranges rather than their true ages because our experience indicate that participants are reluctant to disclose their real ages in research in Nigeria. Participation was voluntary with freedom to withdraw at any time without any negative consequence.

We collected data in three-time periods to minimise biases that may emanate when data for antecedent and outcome variables are collected at the same time from the same source [45]. At Time 1 (T 1), we collected data on participants’ demographics and ethical leadership. After an interwave interval of 2 weeks (T 2), we collected data on perceived leader integrity. The last data were collected for ethical climate after another 2 weeks (T 3). Only participants that participated in the preceding wave of data collection were eligible to participate in the subsequent wave. Given the nature of data collection, we asked those that participated in T 1 to include any form of identification on the questionnaire which was used to identify them at T 2 and T 3. A similar approach was successfully used to collect data in recent studies (e.g., [1, 29]). All completed copies of the questionnaire were returned directly back to us, after which we verbally thanked the participants for their participation. Three hundred and forty-seven teachers participated from T 1 to T 3 out of the 379 teachers who initially agreed to participate. Of these 347 teachers who completed the three set of questionnaires (i.e., T 1, T 2, and T 3), 336 were used for data analysis. The remaining 11 were discarded due to improper completion. Data collection lasted from January to April, 2018.

### Data analysis

First, we conducted structural equation modelling (SEM) using Analysis of Moment Structures (AMOS) version 20 to test whether the hypothesized model fits the observed data. Then we estimated the internal consistency reliability (Cronbach’s α) of the scales, computed descriptive statistics (means and standard deviations), and correlations among the study variables using Statistical Package for the Social Sciences (SPSS) version 21. Next, we used ordinary least squares (OLS) regression-based path analysis based on 5000 bias-corrected (BC) bootstrapped samples to test the hypotheses. The OLS was performed with PROCESS for SPSS macro version 2.13.2 [25]. It allows testing mediating hypothesis at once rather than using separate regression to test it. In this analysis, mediation exists if the BC confidence interval (CI) is entirely different from zero.

## Results

The model adequacy was assessed using goodness-of-fit indices: the root mean square error of approximation (RMSEA), non-normed fit index (NNFI), and comparative fit index (CFI). RMSEA values below .05 indicate good fit, whereas values up to .08 indicate acceptable fit [12]. NNFI and CFI values of .95 are judged as reflecting a good fit, whereas values of .90 represents acceptable fit [12, 26]. Although, the results of the SEM revealed that the chi-square was significant (*x*^2^ = 1924.64, *df* = 17) = *p* < .001) due to the sample size [9], other fit indices, however, showed that the hypothesized model fitted adequately to the data (NNFI = .98, CFI = .98, RMSEA = .02).

Table 1 reports the means, standard deviations, and correlations among the variables. None of the demographic variables (i.e., gender, marital status, job position, education, and age) was significantly correlated with ethical leadership. Among the demographic variables, only gender (*r* = −.12, *p* = .023) and job position (*r* = .12, *p* = .034) had significant negative and positive correlations with perceived leader integrity, respectively. These suggest that female teachers are less likely to perceive their leader (e.g., head teacher) in their workplace as someone with integrity as compared to their male counterparts, and also senior staff members are more likely to perceive their leader in their workplace as someone with integrity in comparison with junior staff. Marital status was the only demographic variable that had a significant negative correlation with ethical climate (*r* = −.13, *p* = .014), indicating that those who are married are less likely to perceive ethical climate in their organisation than their unmarried workmates. Ethical leadership (*r* = .42, *p* < .001) and perceived leader integrity (*r* = .25, *p* < .001) were significantly and positively correlated with ethical climate. The correlation results provide preliminary evidence for confirmation of the hypotheses. The results of the OLS regression-based path analysis used for testing the hypotheses are summarised in Table 2.

**Table 1:** Means, standard deviations, and correlations

	Variable	*M*	*SD*	1	2	3	4	5	6	7
1	Gender	–	–	–						
2	Marital status	–	–	–	–					
3	Job position	–	–	−.17^**^	.40^***^	–				
4	Education	–	–	−.04	.31^***^	.53^***^	–			
5	Age	–	–	−.07	.40^***^	.37^***^	.29^***^	–		
6	Ethical leadership	38.09	8.17	.06	.01	.07	.08	−.02	–	
7	PLI	107.98	17.64	−.12^*^	−.01	.12^*^	.02	.01	.25^***^	–
8	Ethical climate	67.72	10.96	−.10	−.13*	−.06	−.01	.02	.42^***^	.25^***^

*N* = 336, * = *p* < .05 (2-tailed), ** = *p* < .01 (2-tailed), *** = *p* < .001 (2-tailed). *PLI* Perceived leader integrity. Gender was coded 0 = male, 1 = female; marital status: 0 = single, 1 = married; job position: 0 = junior staff, 1 = senior staff; education: 1 = teacher training, 2 = secondary school, 3 = OND/NCE, 4 = HND/bachelor degree, 5 = postgraduate degree, such that higher scores indicated higher educational qualification. Age was coded 1 = < 25 years, 2 = 25–40 years, 3 = 41–56 years, 4 = > 56, with higher scores representing older age

**Table 2:** Simple mediation from ethical leadership to perceived leader integrity to ethical climate

Pathway	Estimate	SE	BC 95% CI		*P*
			Lower	Upper	
EL ➝ PLI	.552	.114	.328	.776	< . 001
PLI ➝ EC	.091	.031	.030	.153	= .004
EL ➝ EC	.529	.067	.396	.662	< . 001
EL ➝ PLI ➝ EC	.050	.026	.005	.106	

BC bootstrapping results were based on 5000 bootstrapped samples

*SE* Standard error, *BC* Bias corrected, *CI* Confidence interval, *EL* Ethical leadership, *PLI* Perceived leader integrity, *EC* Ethical climate

In the OLS regression-based path analysis that appeared in Table 2, gender, marital status and job position were statistically controlled while testing the hypotheses given that these three demographic variables were significantly correlated with perceived leader integrity or ethical climate as shown in Table 1. Gender was significantly and negatively related to both perceived leader integrity (estimate = − 4.699, 95% CI: − 8.700, −.697, *p* = .021) and ethical climate (estimate = − 2.722, 95% CI: − 5.032, −.412, *p* = .021). These indicate that female teachers are less likely to perceive their leader (e.g., head teacher) in their workplace as someone with integrity and also less likely to see their school as being pervaded by ethical climate as compared to their male counterparts. Marital status and job position were not significantly related to perceived leader integrity and ethical climate. The results also show that there was a direct positive relationship between ethical leadership and perceived leader integrity (estimate = .552, 95% CI: .328, .776, *p* < .001), which confirms *H*_1_. Perceived leader integrity had a direct positive relationship with ethical climate (estimate = .091, 95% CI: .030, .153, *p* = .004), and thus confirms *H*_2_. Ethical leadership had a direct positive relationship with ethical climate (estimate = .529, 95% CI: .396, .662, *p* < .001), which confirms *H*_3_. There was an indirect positive relationship between ethical leadership and ethical climate through perceived leader integrity (estimate = .050, 95% CI: .005, .106). Thus, the relationship between ethical leadership and ethical climate was mediated by perceived leader integrity given that the CI was completely different from zero, therefore *H*_4_ was confirmed.

## Discussion

This study aimed to add to the literature on ethical leadership and ethical climate by examining perceived leader integrity as the mechanism underlying this relationship. The study provides evidence in confirmation of *H*_*1*_ that ethical leadership was positively related to perceived leader integrity. This means that the more teachers report that their head teacher demonstrates ethical leadership style, the more likely such teachers also perceive the head teacher as someone high in integrity. This finding reflects the importance of leadership on subordinates’ perception of the leader. That is, the more head teachers walk their ethical talk, the more subordinate teachers are likely to rate such head teachers high in terms of integrity.

In line with *H*_*2*_, the results also showed that perceived leader integrity was positively related to ethical climate. This suggests that subordinates who perceive their head teacher as someone with integrity are also likely to see their workplace as being pervaded by high ethical climate. This could be because such perception of leader integrity may signal to the subordinate teachers that anything short of integrity are not condoned, which may make them perceive the climate of their school as highly ethical.

There was also evidence in confirmation of *H*_*3*_, showing that ethical leadership was positively related to ethical climate. That is, leaders who strive to communicate high ethical values both in words and in deeds by using reward and punishment to elicit ethical conduct from subordinates may covey to teachers that unethical conducts are not condoned in the organisation. With such a leader, the teachers are likely to perceive that ethical climate pervades their school. This finding seems to align with the general tenets of the social learning theory [7], that suggests that individuals model the behaviour of credible leaders in their environment. This finding also supports those of Neubert et al. [41], Mayer et al. [35], Shin [49], and Demirtas and Akdogan [16] who found that ethical leadership was positively related to ethical climate. Therefore, this finding may be generalised to non-teaching contexts such as technology, financial, legal, manufacturing, medical among others, where similar findings have been reported among different climes in previous studies.

Consistent with *H*_*4*_, perceived leader integrity also played a mediatory role between ethical leadership and ethical climate. This indicates that the influence of ethical leadership on ethical climate could be attributed to the role played by perceived leader integrity as the underlying mechanism facilitating this process. That is, perceived leader integrity helps in transmitting the influence of ethical leadership on teachers’ perception of ethical climate. When head teachers place high importance on ethical matters by leading by example as reflected in their behaviour, subordinate teachers are likely to perceive that such leaders have integrity. In turn, the perception of integrity is likely to spawn a general impression from the subordinate teachers that the climate that pervades their school is highly ethical. To our knowledge, this is the first study that provides new evidence showing that perceived leader integrity mediated the relationship between ethical leadership and ethical climate.

## Limitations, strengths, and directions for future research

Some limitations should be highlighted in this study. First, while the current study included only perceived leader integrity as a mediator of the influence of ethical leadership on ethical climate, there are other variables that may influence ethical climate. Perhaps trust in leader is particularly missing from the model tested in our study. For instance, a head teacher who is ethical in performing his or her leadership role may deservedly win the trust of the subordinate teachers which may subsequently influence their perception of the degree of ethical climate in their school. Other variables that have the potential to intervene in the relationship between ethical leadership and ethical climate include: moral competency, moral identity, leader-member exchange, among others. Future research might examine whether these variables serve as the underlying mechanisms and boundary conditions of the influence of ethical leadership.

Second, we studied a small number of homogeneous sample size of teachers that may not be representative of the general population of teachers within Nigeria. Thus, we are unable to generalise from the current results beyond the specific sample used in this study. Although, the use of homogeneous sample appears to enhance the internal validity of our results, we encourage future research to use larger number of heterogeneous samples from different organisations in order to enhance generalisation.

Finally, given that all our data were gathered from the same source, there is possibility that our results may have been contaminated by same source bias. Same source bias might be avoided in future research by obtaining data from different sources based not only on self-report and observer report, but also on subjective as well as objective assessments. Nonetheless, we tried to reduce the problem associated with same source bias and common method variance by adopting procedural remedies [45]. For the procedural remedies, we collected data by allowing time lag between the antecedent, mediator, and criterion variable, assured the participants of the confidentiality of their responses, separated the scales used for measuring the variables, used different response format for the scales and encourage them to respond honestly.

## Implications of findings

The findings of this study have important theoretical and practical implications for the organisational ethics literature and stakeholders in the education sector. First, the observation that ethical leadership was significantly linked to both perceived leader integrity and ethical climate has some implications. Given that leaders play a major role in creating and maintaining ethical or unethical climate [47, 60], ethics training for head teachers based on the tenets of social learning theory might be beneficial in increasing perceived leader integrity and ethical climate. Mayer et al. [36] enthuse that such training might include topics such as “communicating the importance of ethics, rewarding and supporting employees who behave ethically, [meting out appropriate punishment to those who behave unethically] and serving as ethical role models” (p. 10). Also, during selection process, schools may test for integrity, moral standards, and concern for others with the use of integrity tests, structured interview questions, and in-basket exercises that are designed to tap ethical dilemmas which may increase the chances of selecting ethical leaders into an organisation [36].

Lastly, all the findings in this study also have implications for the reputation of a school especially for job applicants and job incumbents. This stems from the fact that the decision on whether or not to work for a particular organisation by job applicants is based in part on ethical concerns [50, 58]. Thus, a school characterised by unethical practices might not attract teachers that are ethically upright in their dealings and will portray the school in a bad light. However, if the school is characterised by ethical practices, it could attract teachers with high ethical conduct. Hence, head teachers and their subordinates in schools might want to consider boosting their ethical image by maintaining high ethical standards in their dealings and thus, attracting ethically upright teachers. For job incumbents, perceiving their head teacher as someone who has integrity might impact on their own behaviour such that they may want to emulate the head teacher and at the same time perceive that they are being surrounded by an ethical climate. Such incumbents are also likely to convey the image of the school to the society. However, the type of image conveyed will depend in part whether the school operates on ethical or unethical foundation.

## Conclusion

The current study extends the social learning theory [7] by identifying perceived leader integrity as the process underlying the relationship between ethical leadership and ethical climate. Several conclusions can be drawn from the findings of this study. First, the study provides support for ethical leadership as an important factor in teachers’ perception of their leaders’ integrity. Second, the study also evinced that perceived leader integrity was positively related to ethical climate. Third, ethical leadership was positively related to ethical climate. One can say with high level of certainty that the finding that ethical leadership was positively related to ethical climate may be generalised to non-teaching contexts such as, financial, legal, manufacturing, management, engineering, medical among others, where similar findings have been reported among different climes and occupational/organisational contexts (e.g., [16, 35, 49]). And lastly, perceived leader integrity mediated the positive relationship between ethical leadership and ethical climate. Thus, in addition to other studies (e.g., [48, 60]) that provided evidence for moderating role of perceived leader integrity and related constructs in influencing ethical climate, the current study suggests that perceived leader integrity can also mediate the ethical leadership–ethical climate relationship. As such, a head teacher who demonstrates ethical behaviour can be seen by the subordinates as someone with integrity which in turn influences their perception regarding the ethical climate in their school. This study therefore offers insights relevant to ethical conducts in schools.

Finally, although not hypothesized, the results of the current study also indicate that gender may play a role in teachers’ perception of their head teachers’ integrity and ethical climate of their school. However, these were contrary to earlier studies that showed no significant difference between male and female employees of manufacturing firms within the United States in rating their supervisors’ integrity (e.g., [38]) and no significant difference between male and female nurses in Israel in their perception of ideal ethical climate (e.g., [21]). Although the samples in these studies differed from those of the current study, more studies involving different occupational groups are, however, needed to substantiate and explicate the role of gender in perception of leader integrity and ethical climate before a firm conclusion can be reached regarding the role of gender.

## Data Availability

The datasets generated and/or analysed during the current study are not publicly available because permission was not obtained from participants to share their data publicly but are available from the corresponding author on reasonable request.

## Abbreviations

OLS: Ordinary least squares
FCT: Federal Capital Territory
OPEC: Organization of Petroleum Exporting Countries
EFCC: Economic and Financial Crimes Commission
ICPC: Independent Corrupt Practices and other Offences Commission
JAMB: Joint Admissions and Matriculation Board
CBT: Computer-Based Test
UTME: Unified Tertiary Matriculation Examination
WAEC: West African Examinations Council
CER: Cancellation of entire results
CEOs’: Chief executive officers’
OND: Ordinary national diploma
NCE: National certificate of education
HND: Highest national diploma
T 1: Time 1
T 2: Time 2
T 3: Time 3
ELS: Ethical Leadership Scale
PLIS: Perceived Leader Integrity Scale
SEM: Structural equation modelling
AMOS: Analysis of Moment Structures
SPSS: Statistical Package for the Social Sciences
BC: Bias-corrected
CI: Confidence interval
RMSEA: Root mean square error of approximation
NNFI: Non-normed fit index
CFI: Comparative fit index
PLI: Perceived leader integrity
SE: Standard error
EL: Ethical leadership
EC: Ethical climate
